# Dissecting infant and maternal antibody repertoires exposes the early onset of infant humoral immunity

**DOI:** 10.1002/cti2.70073

**Published:** 2026-01-11

**Authors:** Albert Bondt, Minjie Tan, Kelly A Dingess, Danique MH van Rijswijck, Yuexiao Chen, Shuai Zhu, Ye Tian, Tianhui Lin, Yuanzhen Zhang, Yanyi Huang, Guanbo Wang, Jing Zhu, Juanjuan Guo, Albert JR Heck

**Affiliations:** ^1^ Biomolecular Mass Spectrometry and Proteomics, Bijvoet Center for Biomolecular Research and Utrecht Institute for Pharmaceutical Sciences Utrecht University Utrecht the Netherlands; ^2^ Netherlands Proteomics Center Utrecht the Netherlands; ^3^ Institute of Chemical Biology Shenzhen Bay Laboratory Shenzhen China; ^4^ Institute of Biotechnology and Health Beijing Academy of Science and Technology Beijing China; ^5^ Department of Gynaecology and Obstetrics Hubei Clinical Research Center for Prenatal Diagnosis and Birth Health, Zhongnan Hospital of Wuhan University Wuhan China; ^6^ Biomedical Pioneering Innovation Center Peking University Beijing China; ^7^ Present address: Danone Global Research and Innovation Utrecht the Netherlands; ^8^ Present address: Mass Spectrometry Core, Changping Laboratory Beijing China

**Keywords:** clonal repertoire, immunoglobulins, infant, mass spectrometry

## Abstract

**Objectives:**

The early development of humoral immunity is important for long‐term protection of the newborn. Here, we set out to discern these infant‐produced antibodies from the vast background of maternal antibodies, which is challenging but essential to shed light on the infant‐produced repertoire.

**Methods:**

Using IgA1 and IgG1 antibody clonal repertoire analysis by mass spectrometry, we compared matched maternal serum, maternal milk, and infant serum samples, both at birth (T1) and at 7–11 weeks after delivery (T2) in four mother–infant dyads.

**Results:**

We observed for both IgA1 and IgG1 unique infant‐produced antibody repertoires at T2. For IgA1 at T2, no substantial clonal overlap was found between infant serum and breastmilk. The serum IgG1 clonal repertoires were highly alike at birth for mother and infant, but at T2, the contribution of the maternal clonal population in the infant had been drastically reduced, and a large portion of the T2 IgG1 repertoire originated from the infant.

**Conclusions:**

Newborns produce their own antibody repertoires as early as a few months after birth. From this small study, no convincing evidence is found for transfer of milk antibodies into the infant circulation.

## Graphical abstract

Can we dissect the newborn's own humoral antibody repertoire from the bulk it receives from its mother through placenta (IgG) or through breastfeeding (IgA)? Yes we can! We show that both IgA1 and IgG1 showed unique, infant‐produced clones at 7–11 weeks after birth, not found in maternal serum or breastmilk.

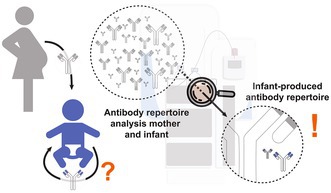

## Introduction

Parents naturally strive to do what is best for their children. Even during pregnancy, many expectant mothers begin taking careful steps regarding dietary intake, substance exposure and physical activity to support foetal development. Luckily, nature supports them in their protective efforts by supplying the unborn foetus with large amounts of maternally produced immunoglobulin (Ig)G antibodies through the placenta to the umbilical cord.^1^ Similarly, large amounts of lgA are delivered to the infant after birth through breastfeeding. A growing body of evidence supports also the transfer of B and T cells from mother to foetus through the placenta,^2^, ^3^, ^4^ contributing to an early—by the mother initiated but by the infant executed—immune response.

Maternal transferred immunity supports the immune naïve newborn while the infant grows and develops its own more mature adaptive immune system. Maternal antibodies indisputably protect the infant against infections, but they may not be purely beneficial in the long term. Different hypotheses exist on how the maternal antibodies suppress the infant's ability to develop long‐term protection after infection or vaccination. Examples of these are via antigen masking, blunting, or interference, thereby dampening the infant's own immune response.^5^, ^6^, ^7^, ^8^ The humoral response must, however, transition from—partially—maternal to fully infant. How and when this transition occurs remains a topic of debate. A major challenge lies in dissecting the maternal and infant contributions to the humoral response. Typically, the distinction is made by studying antibodies in an antigen‐specific fashion, focussing on an antigen that either mother or infant cannot (yet) have encountered. However, activation and multiplication of the cells that were transferred *in utero* or through breastfeeding to the infant may still interfere in discerning whether part of the measured response is already derived from the infant's own immune system.

For optimal timing of, for example, childhood vaccinations, it is important to know when a full response can be induced by the infant. Here, we took on the challenge of dissecting the newborn's produced humoral immune response from the abundant maternal antibody presence. We therefore applied antibody clonal repertoire analysis by mass spectrometry on four mother and infant pairs. We obtained maternal serum and milk and infant serum at two time points, namely T1 at birth and T2 7–11 weeks after delivery. Using this approach, we can qualitatively and quantitatively monitor, with clonal resolution, hundreds of the most abundant antibodies present in a body fluid. Previously, we have shown that there is virtually no clonal overlap in antibody repertoires considering different individuals, and that the repertoire of a healthy adult is highly persistent for several months.^9^, ^10^, ^11^, ^12^ We also demonstrated that there may be substantial overlap between IgA clonal repertoires from milk and serum within a donor.^11^ Here, we hypothesised that, extending upon this earlier work, we should be able to dissect newly infant‐produced antibodies from the vast repertoire of the maternal antibodies using the molecular and clonal resolution provided by the intact LC–MS‐based Fab profiling approach.^9^, ^10^ In addition, if there would be transfer of maternal IgA antibodies from milk to the infant circulation as has been postulated before,^13^ we should be able to detect such maternal clones in the infant's serum.

## Results

### Infant production of immunoglobulin A

**Figure 1:**
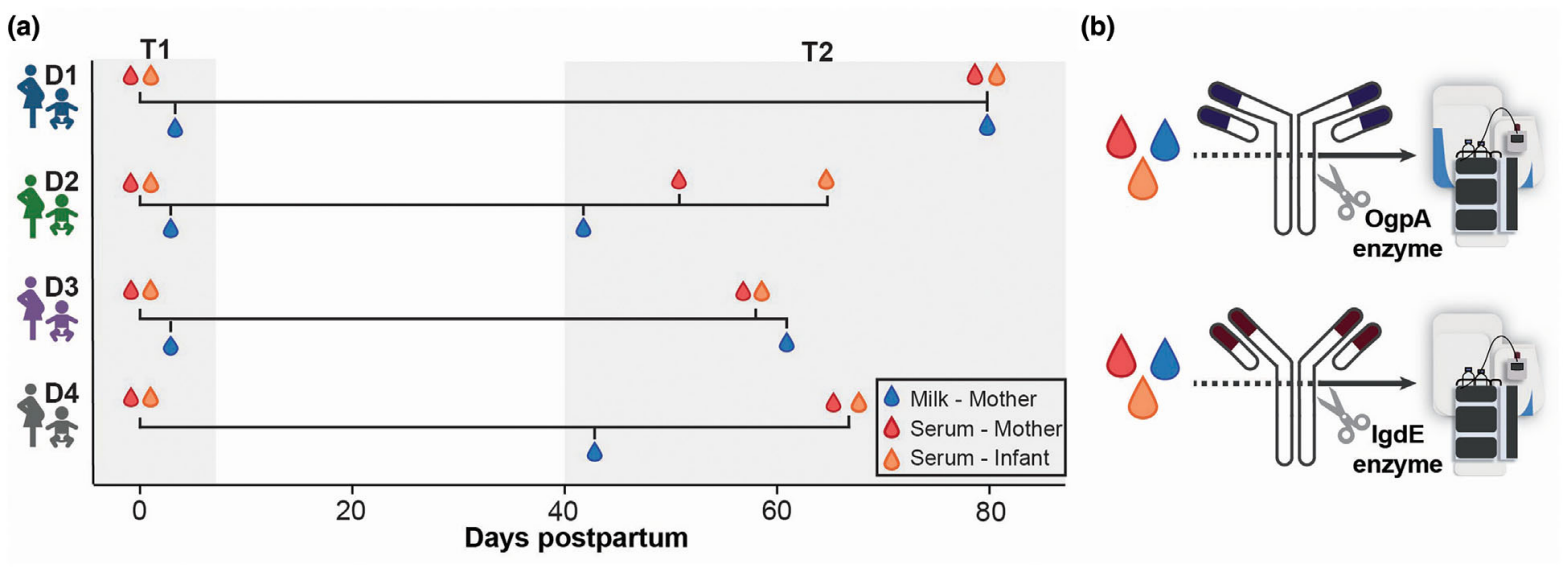
Sample collection and repertoire analysis approach. **(a)** Maternal serum and cord blood were collected at birth, colostrum at day 3 (T1). T2 was collected after 6 weeks or more, as indicated. **(b)** Antibodies (IgA on top, IgG below) were captured from serum and milk, digested with OgpA (IgA) and IgdE (IgG) enzymes, and the antibody Fab repertoire was analysed by intact protein LC–MS.

**Figure 2:**
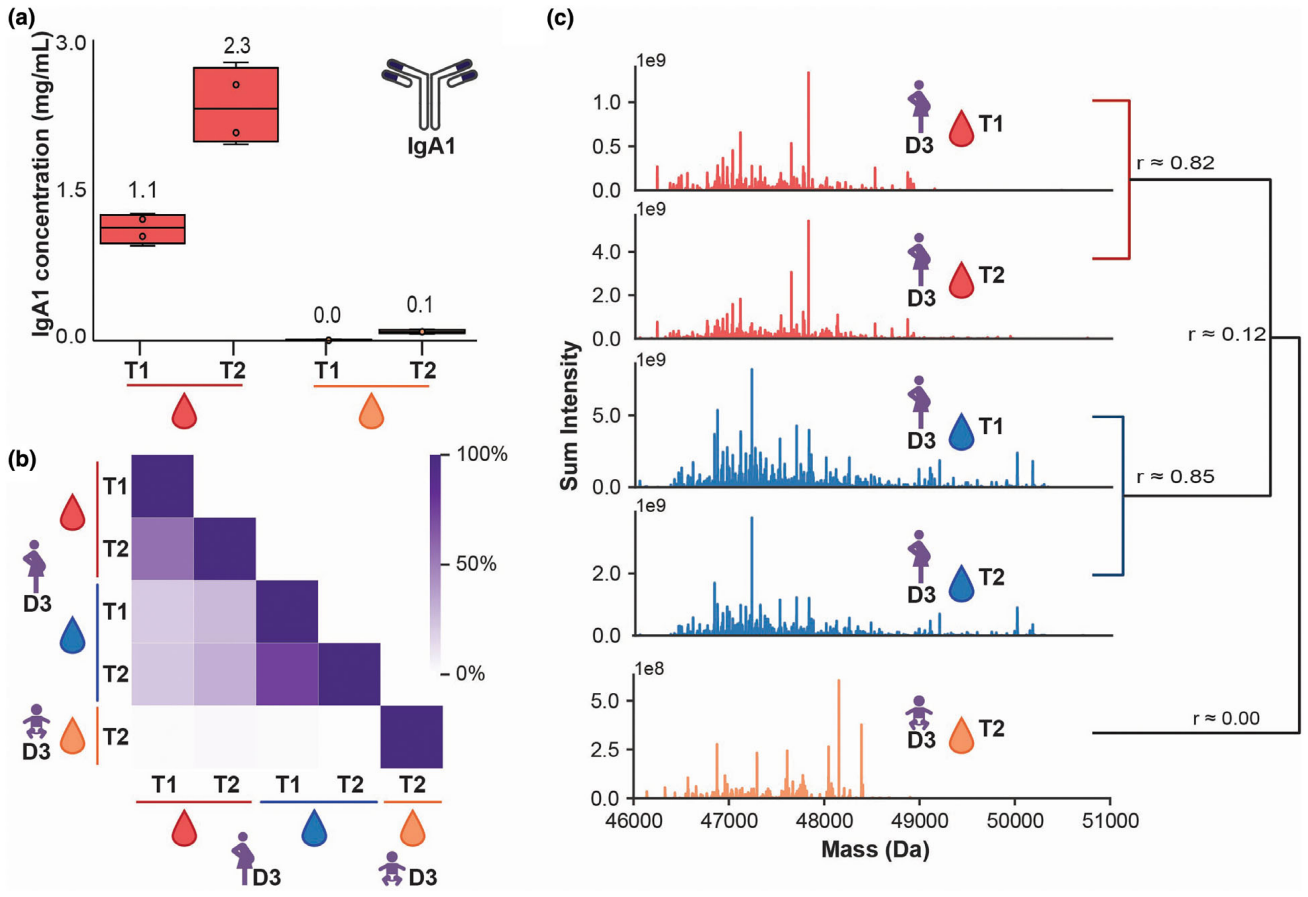
Infant IgA1 is unique and produced early on in the newborn. **(a)** Overall IgA1 concentrations in maternal (red) and infant (orange) serum samples assessed by ELISA. The numerical value represents the median detected concentration. **(b)** Exemplary data from the donor 3 dyad showed substantial clonal overlap in maternal serum (red) and milk (blue) samples—coloured with darker purple as indicated by the scale—but the infant serum sample (orange) is unique. **(c)** A detailed visualisation of the repertoires shows the high similarity between maternal serum (red) and milk samples (blue), whereas the infant repertoire (orange) is distinct. In this representation each vertical line represents a single clone at its detected mass (*x*‐axis), and the *y*‐axis represents the detected intensity. The *r*‐values in the dendrogram at the right further illustrate the dissimilarity of the infant serum.

Maternal and infant samples were collected at birth (serum) or Day 3 (colostrum), and the second time point was collected between 6 and 11 weeks after birth (Figure 1a). First, we determined the overall IgA1 concentrations by ELISA in all collected individual maternal and infant serum samples. As expected, cord blood serum (infant T1) was nearly devoid of IgA (5.6 [4.4–14.7] μg mL^−1^; median [range]), whereas maternal sera showed IgA1 concentrations of 1126.0 [947.1–1266.9] and 2313.3 [1957.0–2774.9] μg mL^−1^ at T1 and T2, respectively (Figure 2a). The lower concentration of maternal serum IgA1 at T1, compared to T2, is likely because of the expected increased blood volume during pregnancy.^14^, ^15^ Milk IgA1 concentrations were 76.4 [25.7–1938.6] and 27.0 [13.7–68.6] μg mL^−1^ at T1 and T2, respectively. The infant sera sampled 7–11 weeks after birth (T2) contained 91.0 [69.8–113.4] μg mL^−1^ of IgA1, a sizeable increase of eight‐ to 20‐fold (Infant 4 and Infant 2), respectively, when compared to at birth, but this concentration is still 10–20 times lower than in the maternal serum.

To investigate the origin of the IgA newly appearing in the infant serum, we next applied antibody repertoire analysis by mass spectrometry (Figure 1b). The low amount of IgA present in cord blood (infant T1) did not allow for the analysis of IgA from these samples, but we could obtain clonal profiles from maternal serum and milk at T1 and T2, as well as from the infant's serum at T2. As previously described in detail,^9^, ^10^ clones were identified based on their unique mass and retention time, and overlap between samples was defined as the observation of the same unique clone identified in the compared samples. We observed a substantial clonal overlap in the mothers' samples within a donor, namely a high overlap between the serum samples at T1 and T2, a high overlap between the milk samples at T1 and T2, and a lower but substantial overlap between serum and milk within a donor (Figure 2b). The IgA1 clones detected in the infant sera at T2, however, were all new and unique; no overlapping clones were observed with any maternal sample (Figure 2b and c, Supplementary figure 1). This indicates that the IgA1 clones in the infant, 7–11 weeks after birth (T2), are not derived from maternal circulation or breastmilk, but are exclusively produced by the infant.

### Infant production of immunoglobulin G

**Figure 3:**
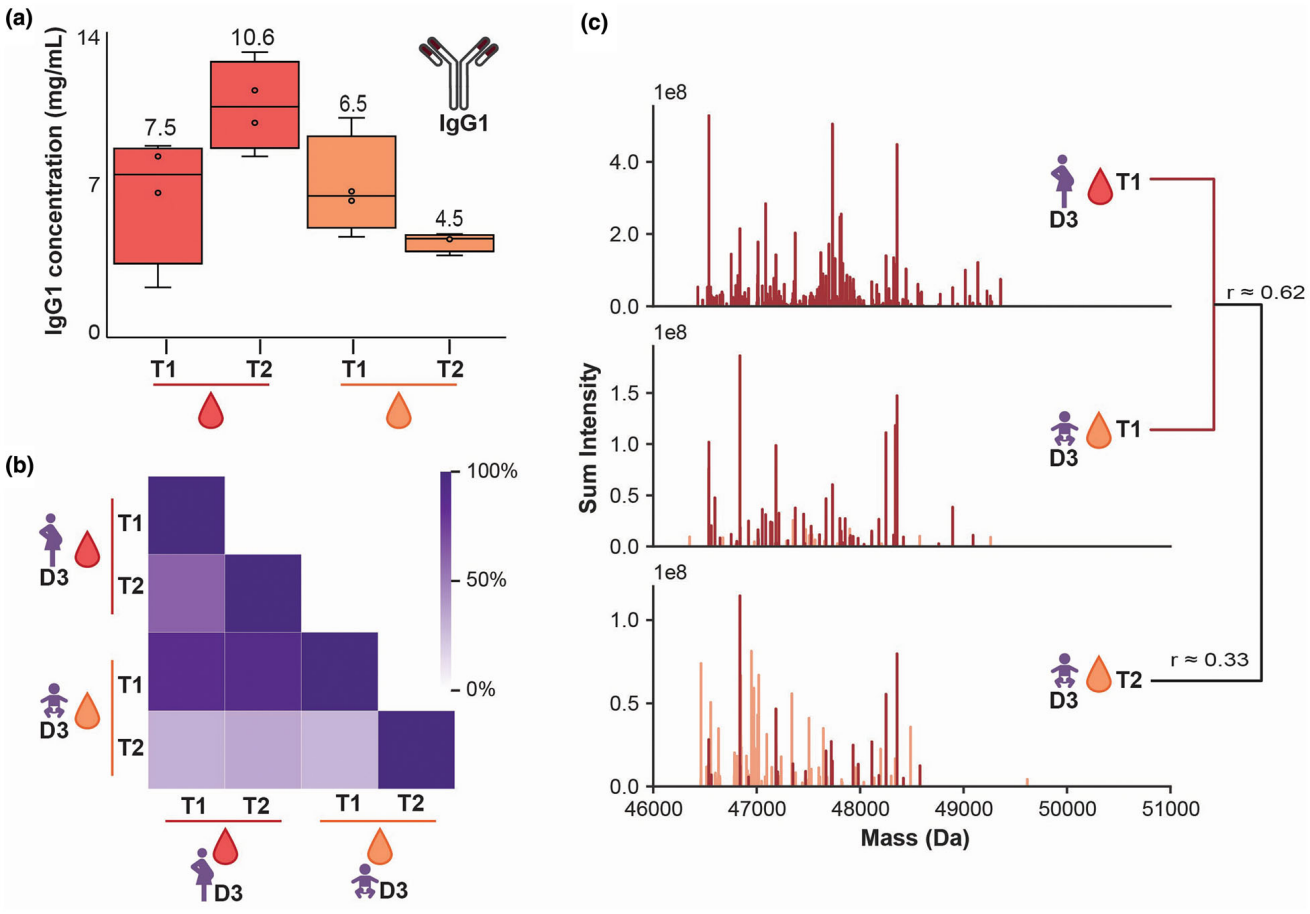
Infant IgG1 becomes populated by unique clones within 7–11 weeks. **(a)** ELISA measurement of IgG1 in maternal (red) and infant (orange) serum samples. The numerical value represents the median detected concentration. **(b)** Substantial clonal overlap was observed between maternal and infant samples as indicated according to the purple scale where darker colour indicates more overlap. The infant sample at T2, however, is starting to deviate. **(c)** A detailed visualisation of the maternal (T1) and infant (T1 and T2) repertoires of the donor 3 dyad shows the abundant presence of maternal clones (red) in infant T1. The infant T2 sample shows lower intensity of the maternal clones, and high abundance of infant‐unique clones (orange). In this representation each vertical line represents a single clone at its detected mass (*x*‐axis), and the *y*‐axis represents the detected intensity. The *r*‐values in the dendrogram at the right further illustrate the lower similarity of the infant T2 serum.

In case of IgA, the detection of unique clones produced by the newborns was straightforward, since infant T1 (cord blood) was largely devoid of IgA (Figure 2a). In case of IgG, however, vast amounts of mothers' IgG are transported to the foetus during pregnancy. This was confirmed by the IgG1 ELISA data, showing alike levels of IgG1 in maternal serum and cord blood (7.5 [2.3–8.8] and 6.5 [4.6–10.1] mg mL^−1^, respectively; Figure 3a). Similar to what we observed for IgA1, the concentration of IgG1 in the mothers' sera at T2 (10.6 [8.3–13.1] mg mL^−1^) was higher than at T1, again likely because of the larger blood volume during pregnancy. Milk IgG1 concentrations were < 0.02 mg mL^−1^.

In infants, a decrease in IgG1 levels is expected to occur over time, since the newborns are cut off from the maternal serum source. Normal IgG1 half‐life is expected to be 3 weeks, but in infants, a half‐life of 7 weeks has been reported.^16^ Based on that, the IgG1 concentration in the infants would be expected to decrease to ~2.7 mg mL^−1^. We indeed did observe a lower IgG1 concentration in the infants, but it was substantially higher than expected, namely 4.5 [3.8–4.7] mg mL^−1^ (Figure 3a). Assuming the half‐life time to be correct, this discrepancy could hint at the infants starting to produce their own IgG1 antibodies.

To test this hypothesis, we next performed antibody repertoire analysis by mass spectrometry, providing a qualitative and quantitative overview of all the most abundant IgG1 clones present in the different samples. As expected, almost exclusively maternal clones were detected in cord blood (Figure 3b, T1), with a high overlap in detected IgG1 clones when comparing samples within a donor dyad, and again no overlap when comparing samples taken from a distinct donor pair. Furthermore, most donors displayed a maternal T2 serum IgG1 repertoire highly similar to T1, even despite a cesarean delivery. In contrast, the infant T2 samples contained a sizable fraction of unique ‘new’ clones, supporting the hypothesis that infants do not only produce IgA1 but also sizeable amounts of IgG1 already 7–11 weeks after delivery (Figure 3b and c, T2). Data for other donors are represented in Supplementary figure 2. The proportion of the T2 repertoire, which was of infant origin, was highly donor dyad dependent and ranged between less than 60% (D4) up to >95% (D1; Supplementary figure 2D).

## Discussion

Here, we provide strong evidence for infants' production of immunoglobulin (Ig)A1 and IgG1 already 2–3 months following birth. For both antibody classes, we detected unique and abundant ‘novel’ clones in the infants' sera that were not detected in their dyad mothers' serum. Moreover, analysing the paired breastmilk also provided no evidence that the IgA1 antibodies in the infant circulation would originate from the mothers' breastmilk.

This does not imply that maternal contribution of antibodies to the infant is not important. In particular, the maternal IgAs help to shape the infant's microbiome and provide a layer of protection in the gut and lungs of young infants already from Day 1.^13^, ^17^, ^18^ We hypothesise, however, that possibly the statement about milk IgA transfer from the gut to the infant bloodstream is based on a misunderstanding. It has been reported that IgA‐bound antigens are taken up by M‐cells in the gut.^19^ The antigen is then presented to the infant's immune system, but this does not directly imply the transfer of IgA molecules to the infants' circulation. One report suggests that maternal IgAs are absorbed based on an increase in infant circulating IgA.^20^ However, others have reported early IgA increases also in formula‐fed infants, as well as the presence of IgA‐positive plasma cells in tonsils.^21^, ^22^ Whether maternally derived IgAs are absorbed and taken up into the preterm infant's circulation remains to be determined.^18^ Overall, more work is required to fully address the remaining questions and gaps in understanding maternal IgA transfer to the infant and the fate of these antibodies. Our presented methodology may prove to be a powerful tool in this process if more samples and larger quantities are available.

The functions of maternal IgA in shaping the microbiome and in providing protection within gut and lungs are fundamentally mucosal. This supports the assumption that milk IgA originates from mucosa, in contrast to circulating IgA.^11^, ^23^ The data presented in this study also indicate relatively limited overlap between breastmilk IgA and maternal serum IgA (Figure 2, Supplemental figure 1). Interestingly, the 10–20% overlap resembles the expected levels of J‐chain coupled dimeric IgA in the circulation. Future studies should consider the entero‐mammary axis when investigating the origin of IgA species.^23^

The observation made here of newly synthesised antibodies of the IgG1 and IgA1 classes in newborns provides strong support for the notion of active T and B cells in young infants.^3^, ^24^ Already in the 1960s, it has been reported that IgG synthesis can occur in the human foetal spleen as early as the 20th week of gestation.^25^ Even more so, work by Gitlin and Biasucci on embryo cultures indicated that IgG synthesis might commence as early as at 12 weeks of gestation.^26^

Although our data provide no evidence for it, it has been suggested that maternal IgGs obtained via breastmilk can be taken up by the infant, whereby the intestinal FcRn supposedly plays a significant role.^27^ That we do not find any evidence for that here may be because of the low abundance of these IgGs in breastmilk.

From the presented work, we observe that the newborn infant is capable of producing their own antibodies shortly after birth. Why then does vaccination not work well in very young infants? Why is long‐term protection not yet induced? One potential answer to that question is the weak capacity of the infant bone marrow to host long‐lived plasmablasts.^28^ Of note, this finding has been observed in murine analyses, but not (yet) in humans. Alternatively, germinal centre formation or functioning can be the cause of a lack of long‐term responses. In mice, it has been shown that, although being formed, germinal centres did not induce memory and plasma B cells.^7^ The authors suggested that this was the consequence of the abundant presence of maternal antibodies in infant circulation. More recent evidence shows that antibody responses can be elicited upon longer exposure to vaccine antigens.^29^ Yet another manner to resolve the issue is to work with more potent adjuvants to elicit a better response.^30^ The latter would, however, not overcome the poor survival support in bone marrow. Supposedly, memory B cells are already formed early on.

The here presented antibody clonal repertoire analysis by mass spectrometry could be instrumental in investigating vaccine‐induced responses in newborns. Analysis of mutation rates could highlight to which extent germinal centres are involved in the immune response. Furthermore, using our technology, we could for example study how the immune response in the infant is affected by maternal vaccination.

To end with a critical note, as sampling on newborns is quite invasive, we had to limit our cohort to just four mother–infant pairs. Ideally, this study would be repeated on a larger sample set. Still, the general observations made and conclusions drawn about the onset of the newborn's personal IgG and IgA1 repertoires were robustly observed in all four mother–infant dyads.

## Methods

### Sample collection

Human milk samples were collected from four maternal donors and their respective four newborn babies. This study was approved by the Medical Ethics Committee of Zhongnan Hospital of Wuhan University (approval no. 2020031). For milk samples, colostrum was collected within 3–4 days postpartum, and the mature milk was collected 42–80 days postpartum. For the blood samples, T1 maternal serum and cord blood were collected right after postpartum. T2 maternal serum and infant serum were collected 51–80 days postpartum. Samples were immediately frozen at −20°C, transferred in dry ice within 24 h and stored at −80°C until thawed for analysis.

### ELISA

The analysis of IgA1 was performed using an ELISA kit (EH3254, FineTest, Hubei, China). Maternal serum samples were diluted between 1:60 000 and 1:40 000; infant serum samples were diluted between 1:2000 and 1:200 in assay buffer according to the manufacturer's instructions. The analysis of IgG1 was performed using an ELISA kit (ab100548, Abcam, Cambridge, UK). Serum samples were diluted into 1:50 000 in assay buffer according to the manufacturer's instructions. The assay plates were incubated with TMB chromogen substrate for 30 min at room temperature. Readouts for both IgA1 and IgG1 were performed on a Varioskan LUX Multimode Microplate Reader (ThermoFisher Scientific, San Jose, CA, USA) at 450 nm. Results were calculated automatically using a 4‐parameter standard curve equation.

### IgA and IgG enrichment and Fab generation

The methods used for either IgA1 and IgG1 profiling by intact Fab LC–MS have been previously described.^9^, ^10^ Briefly, IgA was captured using CaptureSelect IgA affinity matrix (ThermoFisher Scientific) while IgG was captured using CaptureSelect FcXL affinity matrix (ThermoFisher Scientific). The IgA or IgG bead slurries were added to Pierce spin columns with screw and washed with PBS or sodium phosphate buffer, respectively. Following the wash steps, samples were added to the spin columns. For either human milk, blood or serum samples, 50 μL of milk or 20 μL of blood per serum was added onto 40 μL of CaptureSelect IgA slurry. Ten microliters of blood per serum was added onto 20 μL of CaptureSelect FcXL. Samples were then incubated for 1 h while shaking at 750 rpm at room temperature. After incubation, beads captured with IgA or IgG were washed four times with PBS or sodium phosphate buffer, respectively. For the hinge region digestion, IgA1 digestion was performed by adding 50 μL PBS containing 40 U SialEXO and incubating for 2 h at 37°C with continuous shaking at 750 rpm. Then, 1 μL (40 U) of OgpA enzyme was added, and incubation was continued overnight. Following overnight digestion with OgpA, 20 μL of pre‐washed Ni‐NTA agarose slurry (1:1 in PBS) was added to the spin columns and incubated for an additional 30 min. Then, the plug was removed from the column, and the flowthrough, containing the IgA1 Fabs, was collected by centrifugation. IgG digestion was conducted similar to the procedure of IgA, except 50 U FabALACTICA enzyme was used.

### Fab profiling by LC–MS

The LC–MS and data processing approaches as previously described (doi: 10.1016/j.cels.2021.08.008). In short, the collected Fab samples were separated by reversed‐phase liquid chromatography on a Thermo Scientific Vanquish Flex UHPLC instrument, equipped with a 1 × 150 mm MAbPac reversed‐phase analytical column, maintained at 80°C during chromatographic separation. The LC was directly coupled to an Orbitrap Eclipse Tribrid mass spectrometer (Thermo Fisher Scientific). Fab samples were injected as 10 μL and subsequently separated over a 62 min gradient at a flow rate of 150 μL min^−1^. Gradient elution was achieved using mobile phases A (0.1% HCOOH in Milli‐Q water) and B (0.1% HCOOH in CH3CN). The instrument was operating in Intact Protein and Low‐Pressure mode for the acquisition of MS data. Spectra were recorded with a resolution setting of 7500 (@200 m z^−1^) in MS1. Scans were acquired in the range of 500–4000 m z^−1^ with an AGC target of 250% and a maximum injection time set to 50 ms. For each scan, 5 μscans were recorded.

## Author contributions

A.B.: conceptualisation, data curation, formal analysis, project administration, method transfer, writing—original draft, review and editing; M.T.: sample preparation and data collection; K.A.D.: conceptualisation, data curation, writing—review and editing; D.M.H.R.: data curation, formal analysis, visualisation, writing—review and editing; Y.C.: writing—review and editing; S.Z.: writing—review and editing; Y.T.: writing—review and editing; T.L.: writing—review and editing; Y.Z.: resources, writing—review and editing; Y.H.: writing—review and editing; G.W.: conceptualisation, project administration, supervision, funding acquisition, writing—review and editing; J.Z.: conceptualisation, project administration, supervision, funding acquisition, writing—review and editing; J.G.: resources, project administration, supervision, writing—review and editing; A.J.R.H.: conceptualisation, project administration, supervision, resources, funding acquisition, writing—review and editing.

## Conflict of interest

The authors declare no competing interests.

## Supporting information


Supplementary figure 1

## Data Availability

All raw data and data analysis code can be requested from the authors.
