# Supplementary material for: Dissecting infant and maternal antibody repertoires exposes the early onset of infant humoral immunity
Source: Clin Transl Immunology. 2026 Jan 11;15(1):e70073. doi: 10.1002/cti2.70073 (PMC12790936; doi:10.1002/cti2.70073)
Supplement: Supplementary file 1 — Supplementary figure 1 [file CTI2-15-e70073-s001.pptx]

## Slide 1
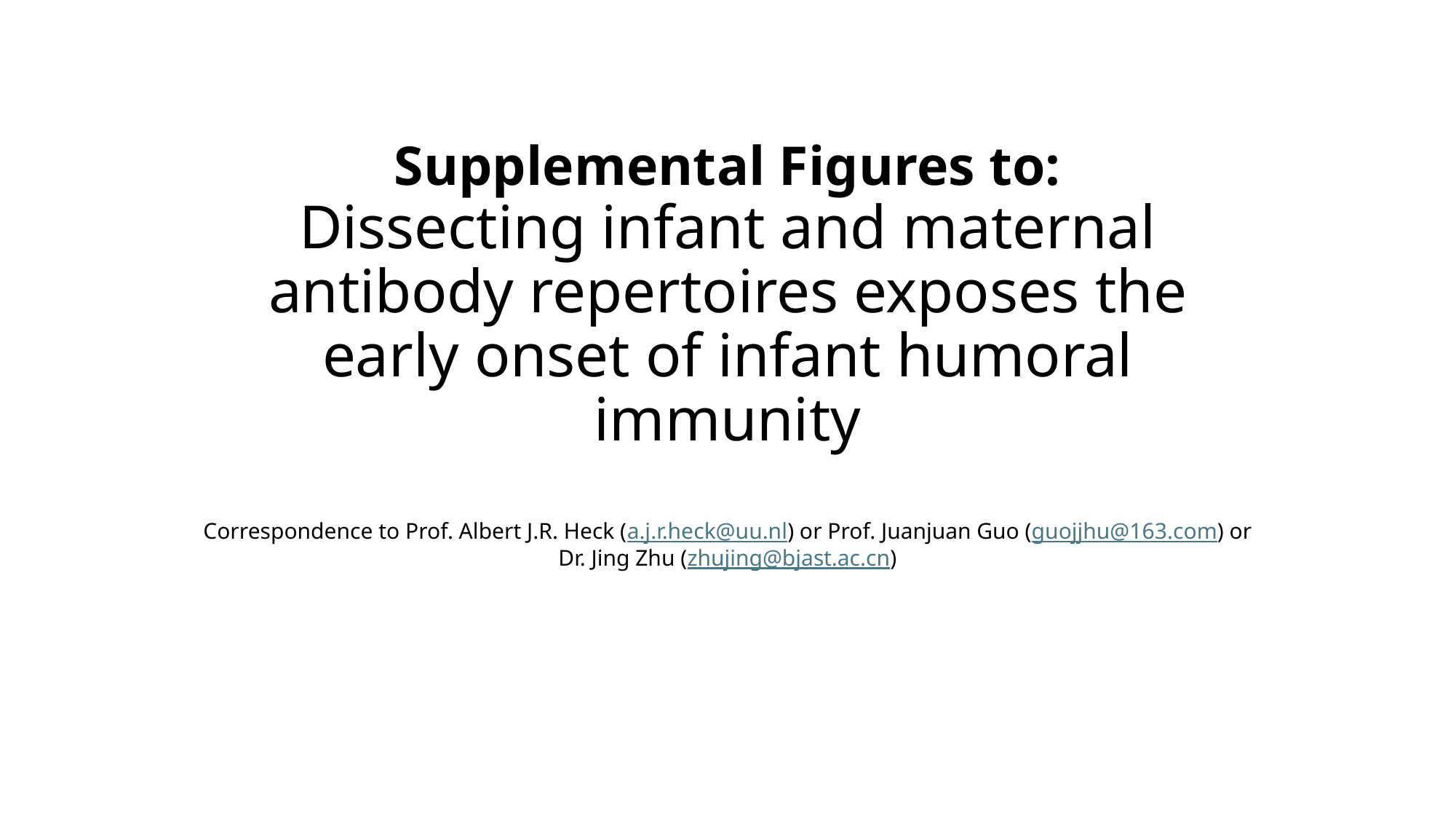

# Supplemental Figures to: Dissecting infant and maternal antibody repertoires exposes the early onset of infant humoral immunity Correspondence to Prof. Albert J.R. Heck (a.j.r.heck@uu.nl) or Prof. Juanjuan Guo (guojjhu@163.com) or Dr. Jing Zhu (zhujing@bjast.ac.cn)

## Slide 2
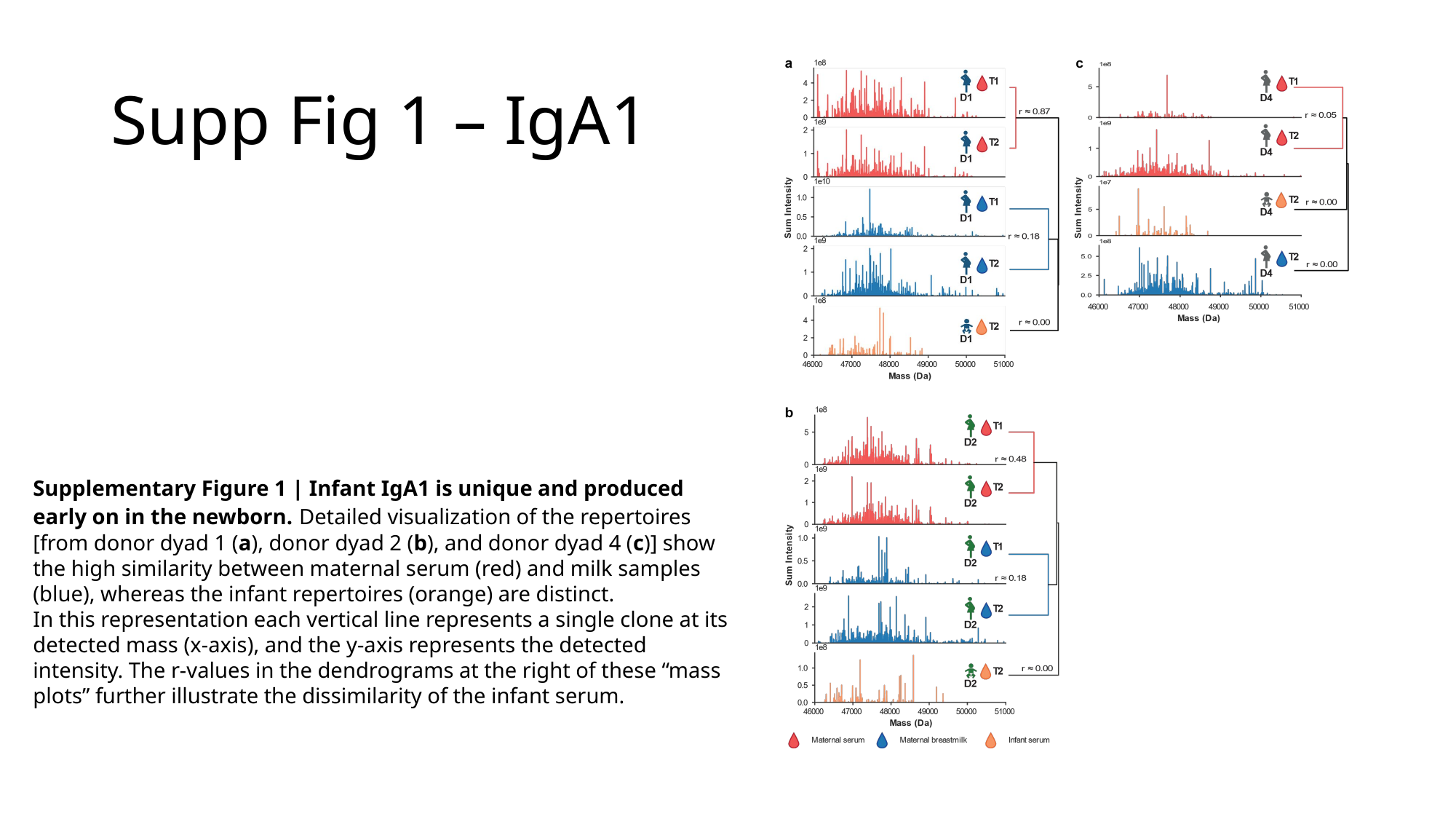

# Supp Fig 1 – IgA1
Supplementary Figure 1 | Infant IgA1 is unique and produced early on in the newborn. Detailed visualization of the repertoires [from donor dyad 1 (a), donor dyad 2 (b), and donor dyad 4 (c)] show the high similarity between maternal serum (red) and milk samples (blue), whereas the infant repertoires (orange) are distinct.
In this representation each vertical line represents a single clone at its detected mass (x-axis), and the y-axis represents the detected intensity. The r-values in the dendrograms at the right of these “mass plots” further illustrate the dissimilarity of the infant serum.

## Slide 3
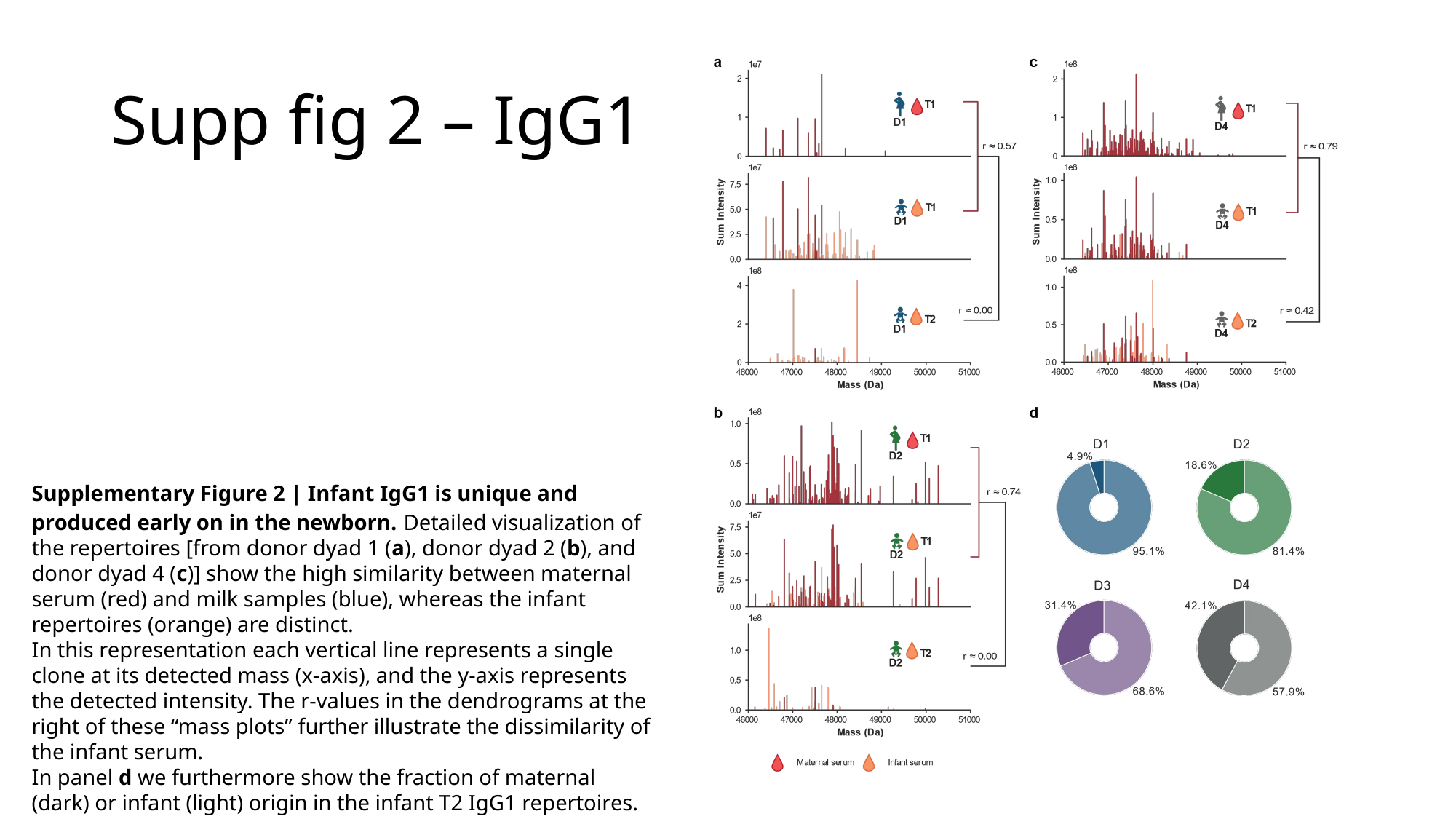

# Supp fig 2 – IgG1
Supplementary Figure 2 | Infant IgG1 is unique and produced early on in the newborn. Detailed visualization of the repertoires [from donor dyad 1 (a), donor dyad 2 (b), and donor dyad 4 (c)] show the high similarity between maternal serum (red) and milk samples (blue), whereas the infant repertoires (orange) are distinct.
In this representation each vertical line represents a single clone at its detected mass (x-axis), and the y-axis represents the detected intensity. The r-values in the dendrograms at the right of these “mass plots” further illustrate the dissimilarity of the infant serum.
In panel d we furthermore show the fraction of maternal (dark) or infant (light) origin in the infant T2 IgG1 repertoires.
